# Supplementary material for: Tethering ferredoxin-NADP+ reductase to photosystem I promotes photosynthetic cyclic electron transfer
Source: Plant Cell. 2025 Mar 3;37(3):koaf042. doi: 10.1093/plcell/koaf042 (PMC11912148; doi:10.1093/plcell/koaf042)
Supplement: koaf042_Supplementary_Data [file koaf042_supplementary_data.zip › Supplemental_figures.pdf]

Supplemental Fig 1

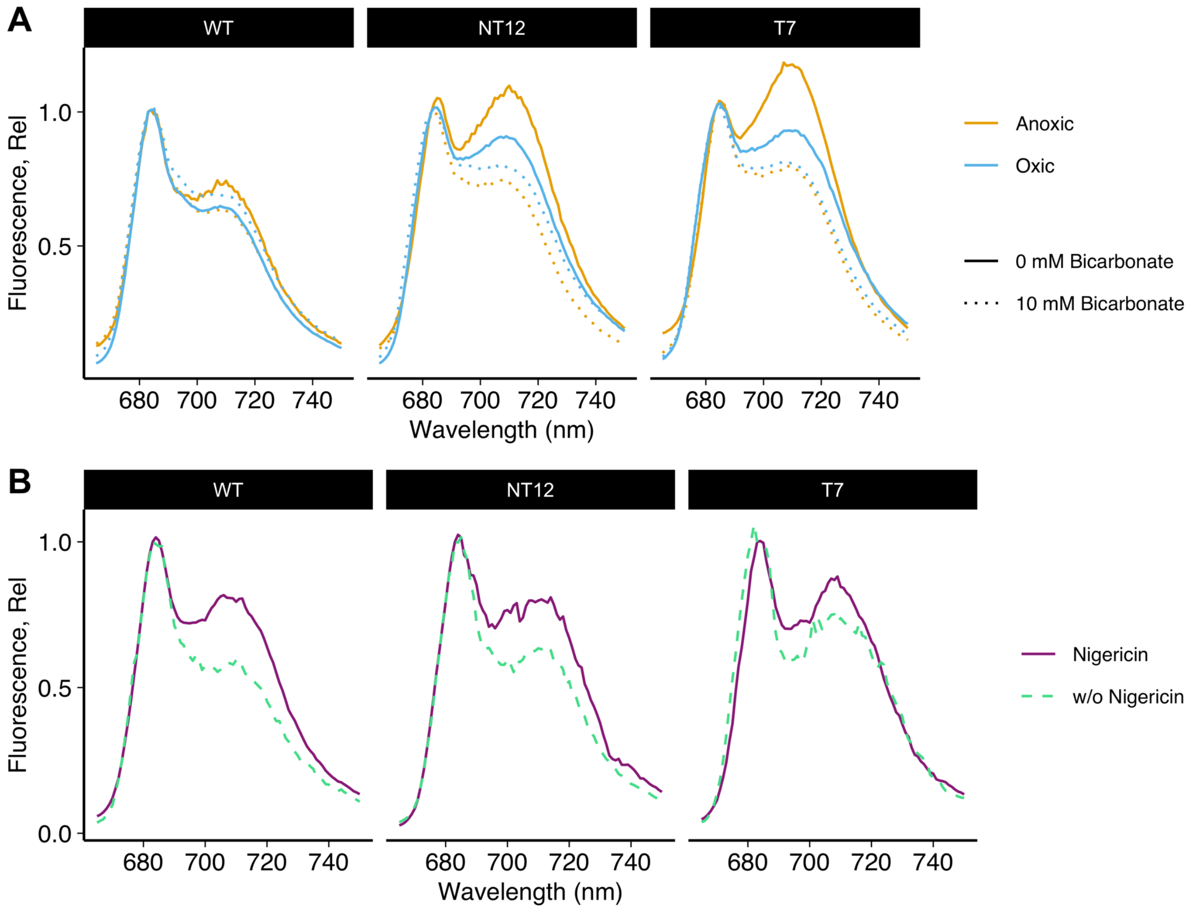

Supplementary Figure S1 | 77K fluorescence emission spectra of WT, NT12 and T7 cells illuminated with 420 nm light. Supports Fig. 3. **A,** Four conditions are shown, with high/ low CO_2 indicating 0 or 10 mM added sodium bicarbonate and oxic/ anoxic indicating with/ without 50 mM glucose, 30 U mL^{-1} catalase and 10 U ml^{-1} glucose oxidase. **B,** Effect of inclusion or omission of 10 μM nigericin on high CO_2/ anoxic cells. Abbreviations: WT, wild-type; NT12, native/tethered line 12; T7, tethered-only line 7.

Supplemental Fig 2

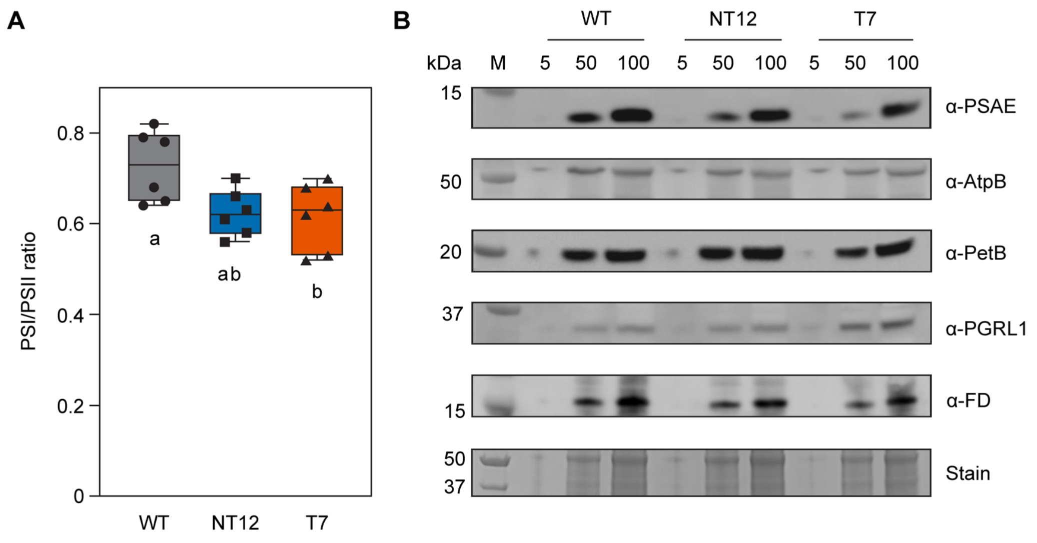

Supplementary Figure S2 | ECS and immunoblot quantification of key proteins expressed in WT, NT12 and T7 cells. Supports Fig. 4. **A,** ECS quantification of PSI/PSII ratio. Median values are plotted as the centre-line in each box (n = 6), box limits are the first and third quartiles, and whiskers show the range. Means were then compared between genotypes using an ordinary two-way ANOVA and corrected using Tukey's multiple comparison test followed by a HSD test with alpha = 0.05. Different letters indicate significant differences between data points. **B,** Immunoblots of key proteins performed on whole cells of WT, NT12 and T7 using antibodies to PSI (PSAE), ATP synthase β subunit (AtpB), cytochrome b_6 (PetB), PGRL1 and FD. Coomassie Stain shows equal protein loading at 5, 50 and 100 µg Chl mL^{-1}. Abbreviations: M, molecular weight marker; WT, wild-type; NT12, native/tethered line 12; T7, tethered-only line 7.

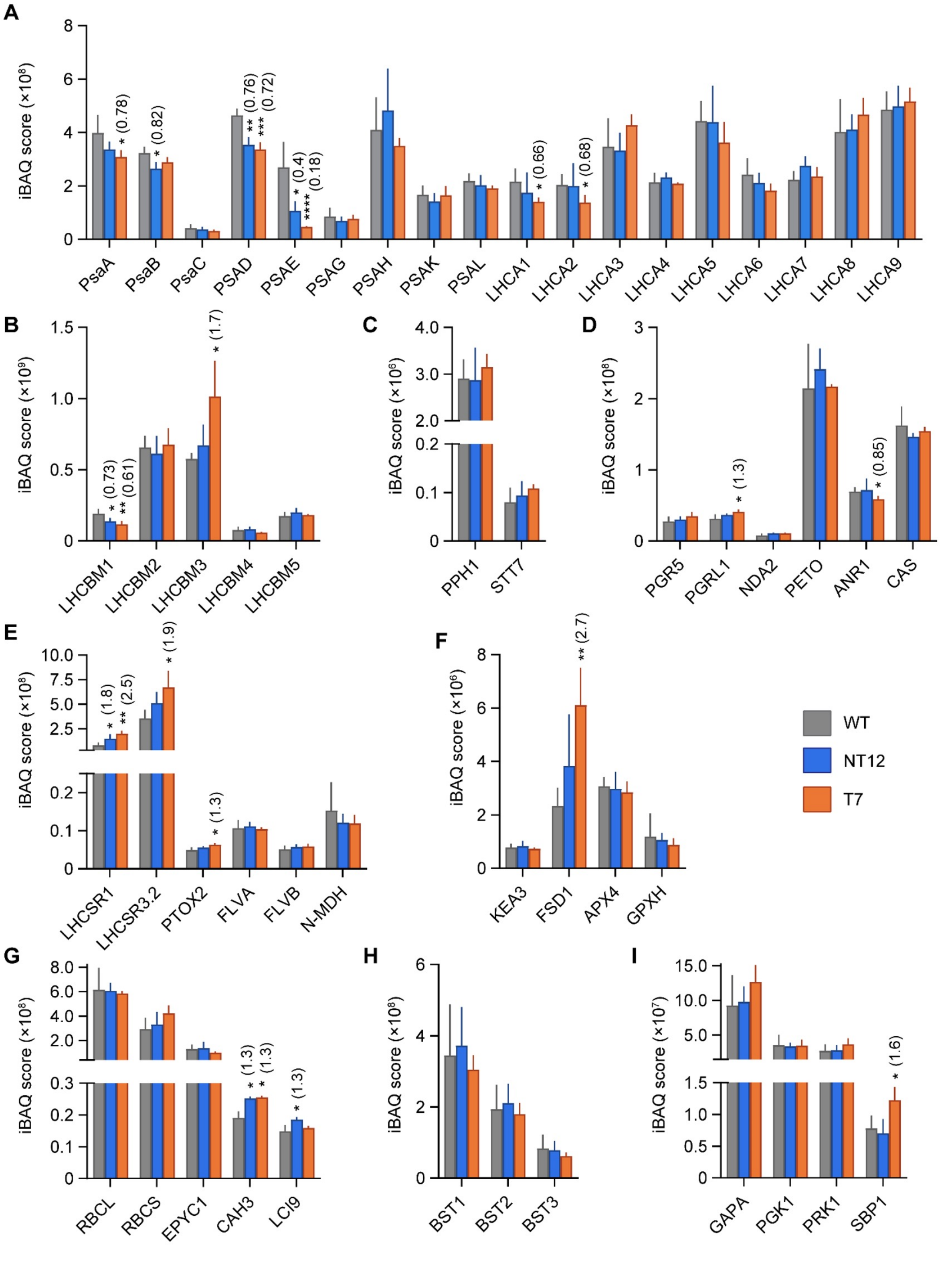

Supplementary Figure S3 | Additional quantification of proteins expressed in WT, NT12 and T7 cells. Supports Fig. 4. Mass spectrometry-based label-free quantification of selected photosynthetic proteins. Bars show means ± SDs. Means in panels A-F are the same as those plotted in the heat maps in Fig 4. **A**, PSI subunits and light harvesting complex I (LHCI) proteins. **B,C**, State transition associated proteins. **D**, Cyclic electron transfer (CET) associated proteins. **E,F**, Stress associated proteins. **G**, Rubisco large (RBCL) and small (RbcS1-2) subunits, essential pyrenoid component 1 (EPYC1), carbonic anhydrase 3 (CAH3) and low CO_2-inducible protein (LCI9). **H**, Bestrophins 1-3 (BST1-3; quantified by the sum of isoform-specific peptide ion intensities). **I**, Glyceraldehyde-3-phosphate dehydrogenase (GAPA), phosphoglycerate kinase (PGK1), phosphoribulokinase (PRK1) and sedoheptulose-1,7-bisphosphatase (SBP1). Note that the y-axes scales vary between panels. *Q* values derived from modified t-tests incorporating permutation-based false discovery rate with 250 randomizations (n = 4) are shown as 0.01-0.05 (*) and 0.001-0.01 (**), 0.0001-0.001 (***) and < 0.0001 (****), with exact q values listed in Supplementary Data Set S1. Fold change relative to WT is shown in parentheses for differences with $q \leq$ 0.05; differences with $q \geq$ 0.05 (not significant) are not shown. Abbreviations: WT, wild-type; NT12, native/tethered line 12; T7, tethered-only line 7.

Supplemental Fig 4

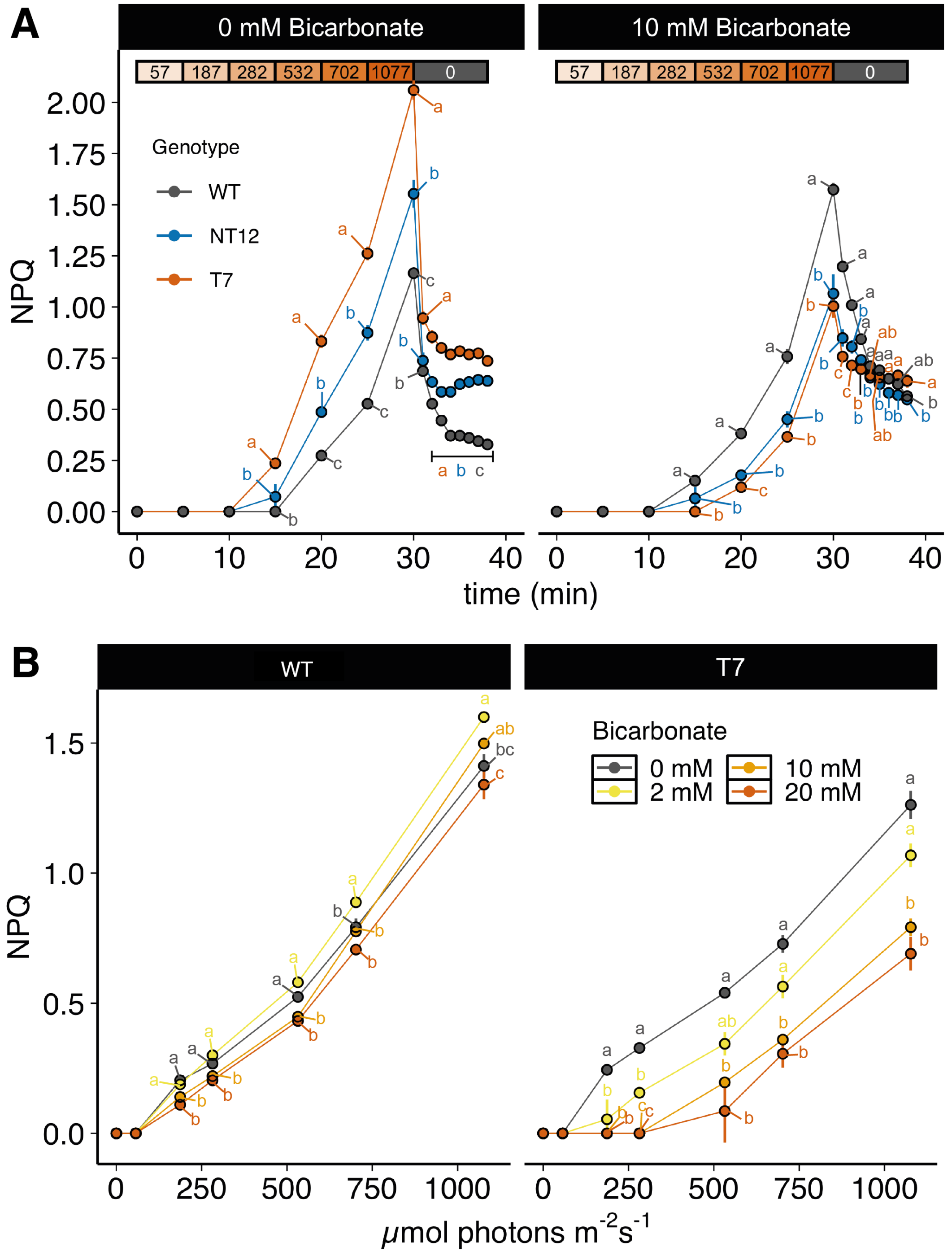

Supplementary Figure S4 | Effect of added sodium bicarbonate on NPQ in oxic cells. Supports Fig. 5. **A,** Effect of 0 or 10 mM added sodium bicarbonate on NPQ recorded at different light intensities (shown in orange bars) over a 30-minute illumination. Relaxation of NPQ in the subsequent dark period. **B**, Light curve of NPQ versus light intensity at different concentrations of added sodium bicarbonate. **C**, Comparison of NPQ of additional T and NT mutants and WT oxic cells without additional sodium bicarbonate. Means were compared between genotypes using an ordinary two-way ANOVA and corrected using Tukey's multiple comparison test followed by a HSD test with alpha = 0.05. Different letters indicate significant differences between data points. Abbreviations: WT, wild-type; NT4 and NT12, native/tethered lines 4 and 12; T7 and T10, tethered-only lines 7 and 10; NPQ, non-photochemical quenching.

Supplemental Fig 5

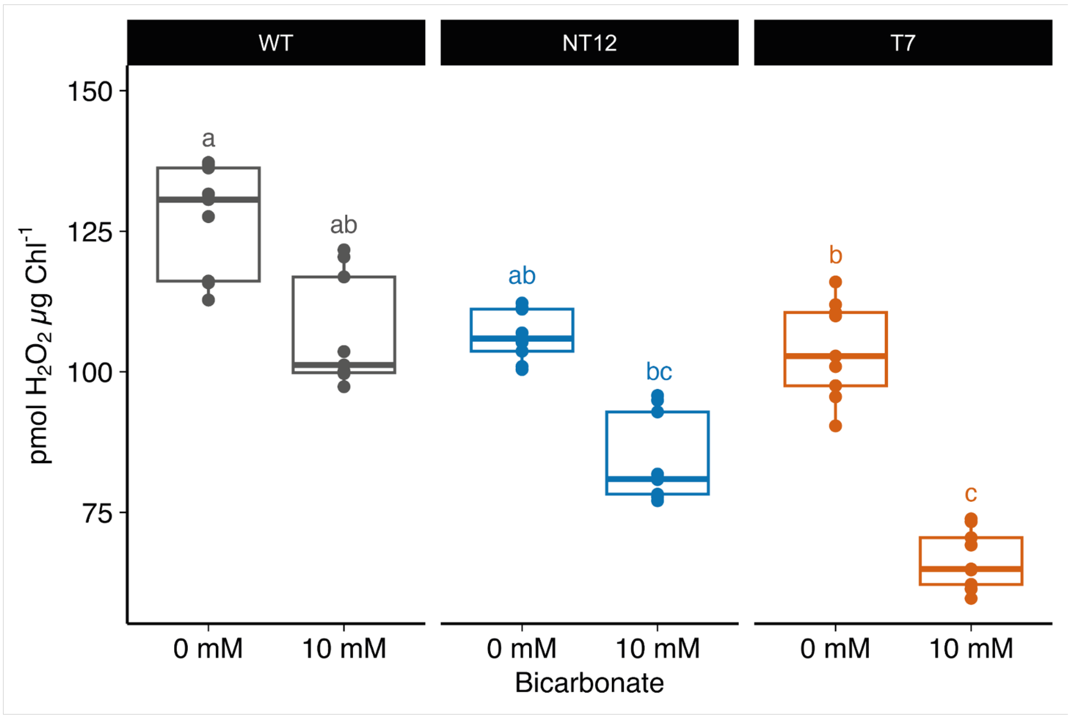

Supplementary Figure S5 | Hydrogen peroxide levels in WT, NT12 and T7 cells. Supports Fig. 7. Cells were grown in tris-acetate-phosphate (TAP) medium and switched to tris-phosphate (TP) medium for at least 24 h. 1 mL of culture was diluted with 1 mL fresh TP containing 1 U of horseradish peroxidase and 5 µM of Amplex Red and incubated under red light at 520 µmol photons m^{-2} s^{-1} for 1 h. H_2O_2 in the supernatant was subsequently quantified using a linear H_2O_2 standard. Box lines represent the median and first and third quartiles; whiskers indicate the range; circles indicate the raw data. Means were compared between genotypes using an ordinary two-way ANOVA and corrected using Tukey's multiple comparison test followed by a HSD test with alpha = 0.05. Different letters indicate significant differences between genotypes and CO_2 concentrations. Abbreviations: WT, wild-type; NT12, native/tethered line 12; T7, tethered-only line 7.

Supplemental Fig 6

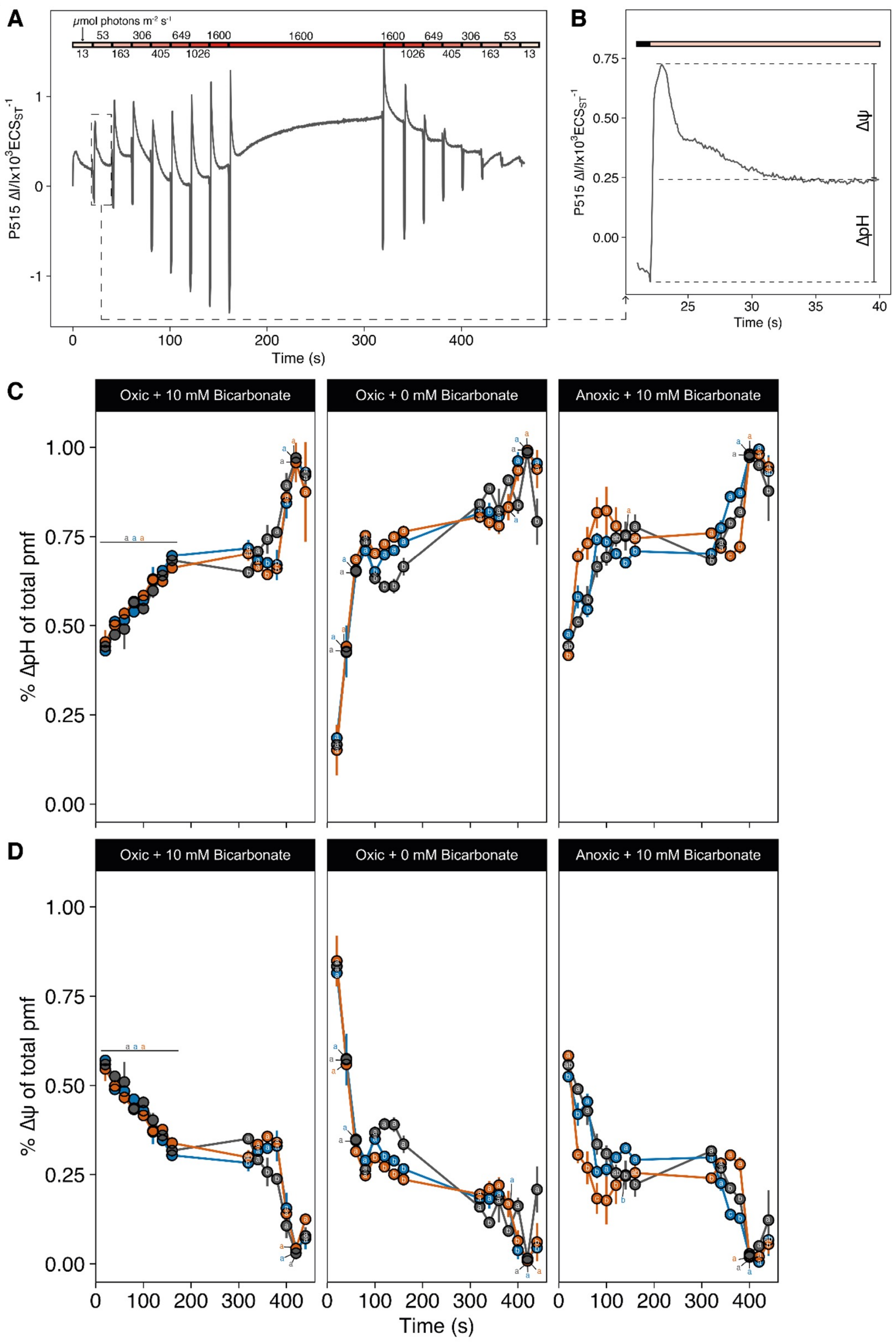

Supplementary Figure S6 | Partitioning of the proton motive force into ΔpH and Δψ. Supports Fig. 6. **A**, Example raw trace of the P515 signal of Chlamydomonas cells. Light intensity was increased every 20 s (shown at the top), followed by a 1.4 s dark pulse. Cells were then illuminated at the highest light intensity for 10 min, followed by a decrease every 20 s. **B**, Illustration of how pmf was partitioned into ΔpH and Δψ. **C**, Percent ΔpH of total pmf under three different conditions in WT (grey), NT12 (blue) and T7 (orange) cells. **D**, Percent Δψ of total pmf under three different conditions in WT (grey), NT12 (blue) and T7 (orange) cells. Means were compared between genotypes using an ordinary two-way ANOVA and corrected using Tukey's multiple comparison test followed by a HSD test with alpha = 0.05. Different letters indicate significant differences between data points. Abbreviations: WT, wild-type; NT12, native/tethered line 12; T7, tethered-only line 7; pmf, proton motive force.

Supplemental Fig 7

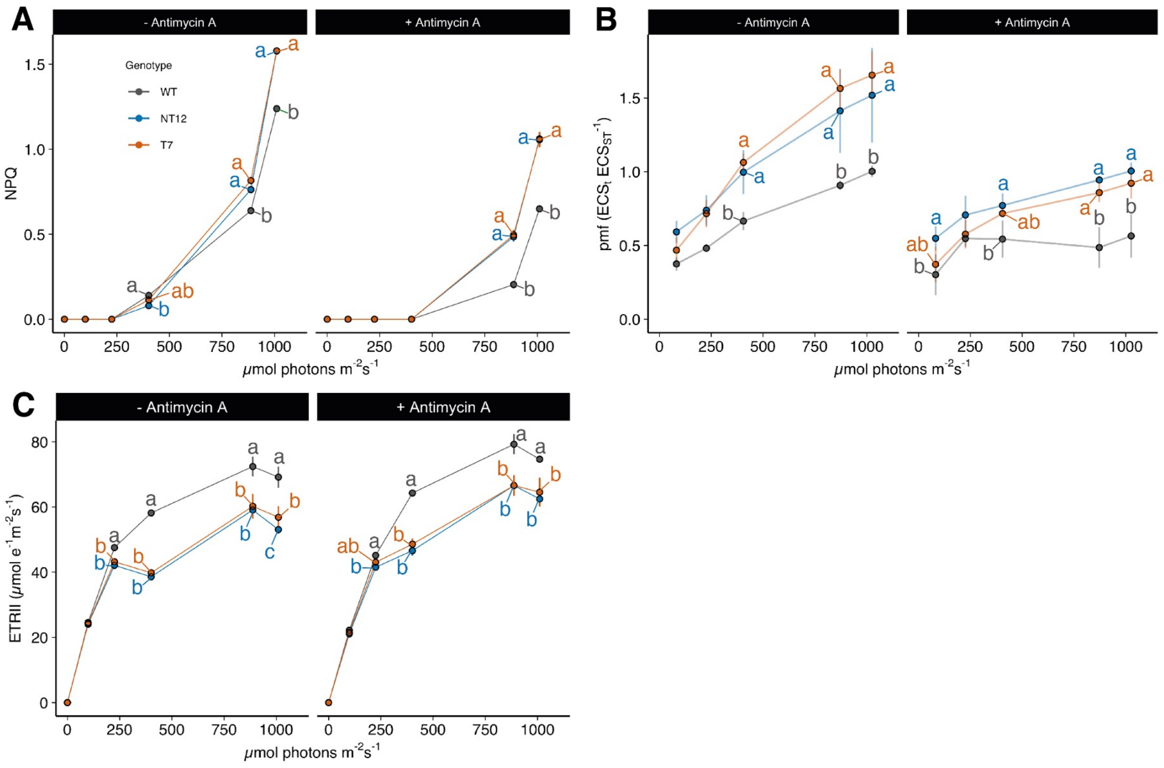

Supplementary Figure S7 | Effect of antimycin A on chlorophyll fluorescence and ECS parameters in WT, NT12 and T7 cells under anoxia with 10 mM $NaHCO_3$. Supports Fig. 9. **A**, non-photochemical quenching (NPQ) ± Antimycin A (AA). **B**, proton motive force (pmf) ± AA. **C**, Electron transport rate (ETRII) ± AA. Measurements were made as described in Figs. 5 and 6 except that 40 µM Antimycin A from a 40 mM ethanolic stock was added prior to measurements. Error bars are S.D. (at least replicates per strain). Means were compared between genotypes using an ordinary two-way ANOVA and corrected using Tukey's multiple comparison test followed by a HSD test with alpha = 0.05. Different letters indicate significant differences between data points at each light intensity. Abbreviations: WT, wild-type; NT12, native/tethered line 12; T7, tethered-only line 7;
